# Lgl2 Executes Its Function as a Tumor Suppressor by Regulating ErbB Signaling in the Zebrafish Epidermis

**DOI:** 10.1371/journal.pgen.1000720

**Published:** 2009-11-13

**Authors:** Sven Reischauer, Mitchell P. Levesque, Christiane Nüsslein-Volhard, Mahendra Sonawane

**Affiliations:** Max-Planck Institut für Entwicklungsbiologie, Department of Genetics, Tuebingen, Germany; University of Pennsylvania School of Medicine, United States of America

## Abstract

Changes in tissue homeostasis, acquisition of invasive cell characteristics, and tumor formation can often be linked to the loss of epithelial cell polarity. In carcinogenesis, the grade of neoplasia correlates with impaired cell polarity. In Drosophila, *lethal giant larvae (lgl)*, *discs large (dlg)*, and *scribble,* which are components of the epithelial apico-basal cell polarity machinery, act as tumor suppressors, and orthologs of this evolutionary conserved pathway are lost in human carcinoma with high frequency. However, a mechanistic link between neoplasia and vertebrate orthologs of these tumor-suppressor genes remains to be fully explored at the organismal level. Here, we show that the *pen/lgl2* mutant phenotype shares two key cellular and molecular features of mammalian malignancy: cell autonomous epidermal neoplasia and epithelial-to-mesenchymal-transition (EMT) of basal epidermal cells including the differential expression of several regulators of EMT. Further, we found that epidermal neoplasia and EMT in *pen/lgl2* mutant epidermal cells is promoted by ErbB signalling, a pathway of high significance in human carcinomas. Intriguingly, EMT in the *pen/lgl2* mutant is facilitated specifically by ErbB2 mediated E-cadherin mislocalization and not via canonical *snail*–dependent down-regulation of *E-cadherin* expression. Our data reveal that *pen/lgl2* functions as a tumor suppressor gene in vertebrates, establishing zebrafish *pen/lgl2* mutants as a valuable cancer model.

## Author Summary

In metazoans, the body surface and linings of several organs are formed from membranous tissue called epithelia. The functions of epithelia include secretion, absorption, and protection. Epithelial cells exhibit polarized distribution of several proteins, which is essential for their function. In carcinomas, which are cancers of epithelial origin, this epithelial cell polarity is impaired. Intriguingly, defects in cell polarization can also lead to tumorigenesis in some animal model systems. It is thus important to understand how cell polarization and epithelial growth control are linked so as to treat the carcinomas better by identifying new drug targets. Here we show that in zebrafish a gene named *lethal giant larvae 2* (*lgl2*), which is essential for the establishment of epithelial cell polarity, also acts as a suppressor of malignant growth properties in the epidermis, an epithelial component of the skin. We further show that in absence of *lgl2* function increased epidermal growth factor receptor activity imparts malignant properties to the epidermal cells. Thus, we report here a mechanism by which epithelial cells acquire malignant characteristics when cell polarity is impaired in absence of *lgl2* function.

## Introduction

Tumor suppression is a concept first formulated in *Drosophila* after emerging evidence that recessive mutations can lead to the formation of cellular overgrowth [1],[2]. To date, more than 50 tumor suppressor genes have been identified in *Drosophila* [3]. Their deficiencies result in benign hyperplasias to malignant neoplasms. Amongst these tumor suppressors, mutations in *lethal giant larvae* (*lgl*), cause malignant neoplasias in imaginal discs and the brain when transplanted into wild type adult host flies [4],[5]. In *Drosophila* neuroblasts, *lgl* function is essential for localization of the cell fate determinant Numb, mislocalization of which in *lgl* mutant larvae prevents the neuroblasts from dividing asymmetrically and therefore causes neuroblastoma [6]–[9]. Furthermore, it has been proposed that *lgl* prevents tumor formation by antagonizing the activation of Dpp signaling by semaphorin 5c in the brain [10]. Although epithelial overgrowth phenotypes have been reported in *lgl* mutant larvae in *Drosophila* [1], the mechanism by which *lgl* manifests its effects on epithelial growth remains to be understood. Nevertheless, it is known that along with *lgl*, two other tumor suppressor genes, discs large (*dlg*) and scribble (*scrib*), primarily act in the maintenance of apico-basal cell polarity in epithelial cells [11].

The establishment as well as the maintenance of apico-basal cell polarity and eventually the depolarization of a cell is a complex process, involving several factors. In recent years, a conserved mechanism for the establishment and maintenance of apico-basal cell polarization has emerged, which mainly involves two pathways. Accordingly, the formation of the apical domain is controlled by the Par (partitioning defective) pathway, which consists of the PDZ domain containing proteins Par3, Par6, and atypical protein kinase C (aPKC). In contrast, a pathway consisting of *disc-large* (*dlg*), *scribble* (*scrib*) and *lethal giant larvae* (*lgl*) regulates the formation and maintenance of the baso-lateral domain [12],[13]. Intriguingly, only mutations in genes that act in the baso-lateral pathway (e.g. *lgl, dlg, scrib*) lead to a neoplastic growth phenotype in *Drosophila* [2], [14]–[16].

The two vertebrate orthologs of the *Drosophila lethal giant larvae* gene have conserved functions in the maintenance of cell polarity and tissue homeostasis. Disruption of *lgl1* function results in the loss of apical junctional complex in neuroblasts and hyperplasia of the brain in mouse [17]. Furthermore, it has been shown for human melanoma cell lines, that a human homolog of *lgl*, *hugl1* is significantly down-regulated. Artificial induction of *hugl1* in these cell lines reduces their migratory potential with concomitant transcriptional up-regulation of the cell adhesion molecule E-cadherin (E-cad) and a down-regulation of *matrix-metalloproteinases* (*mmp*s), both of which are known to be involved in suppression of epithelial-to-mesenchymal transition (EMT), a process which enables an epithelial cell to gain mesenchymal or migratory properties [18]. Recently, a significant correlation between the loss of *hugl1* and a poor clinical prognosis for cancer patients has been shown [19].

The forward genetic approach in zebrafish has revealed a novel function for the second *lgl* ortholog, *pen/lgl2*, in maintenance of the epidermal integrity, which is a stratified epithelium. The *pen*/*lgl2* deficiency primarily results in the loss of hemidesmosomes, cellular junctions that mediate cell-matrix adhesion [20]. It has been shown that Lgl2 localizes to the lateral domain of the epidermal cells and regulates hemidesmosome formation by mediating the targeting of ITGa6, a component of hemidesmosomes, to the membrane [21]. Furthermore, epithelial cells in the *pen/lgl2* mutant exhibit altered epidermal cell morphology as well as cell polarity and enhanced epidermal growth [20],[21]. Recently, it has been shown that in colorectal and breast carcinoma cell lines, a member of the ZFH family of repressors ZEB1 regulates the levels of Lgl2. The loss of ZEB1 function restores Lgl2 levels and the epithelial phenotypes in tumor cells, which suggests that Lgl2 acts as an effector of ZEB1 in tumor suppression [22].

From the analysis of several cancer models it is evident that autocrine self-stimulation with growth factors is one of the hallmarks of tumorigenicity [23],[24]. However, whether activation of growth factor signaling is a consequence of the loss of baso-lateral pathway components remains unclear to date. Moreover, whether *lgl1* and *lgl2* deficient clonal cell populations can promote tumor formation in vertebrate tissues, a typical characteristic of tumor suppressor genes, remains unresolved. Here, we show that *lgl2* deficient clones indeed promote tumor formation in the zebrafish epidermis. Moreover, *pen/lgl2* mutant basal epidermal cells undergo EMT. Using biochemical analysis, chemical inhibitors and genetic interaction studies, we demonstrate that these phenotypes are a consequence of an over-activation of *erbB* signaling involving at least one *erbB* family member, *erbB2*. Our microarray and immuno-histological analysis reveal that activation of *erbB* signaling facilitates EMT by transcriptional up-regulation of key EMT regulators and a reduction in the membrane localization of E-cad, a known suppressor of EMT.

## Results

### *lgl2* functions as a tumor suppressor gene in the zebrafish epidermis

**Figure 1:**
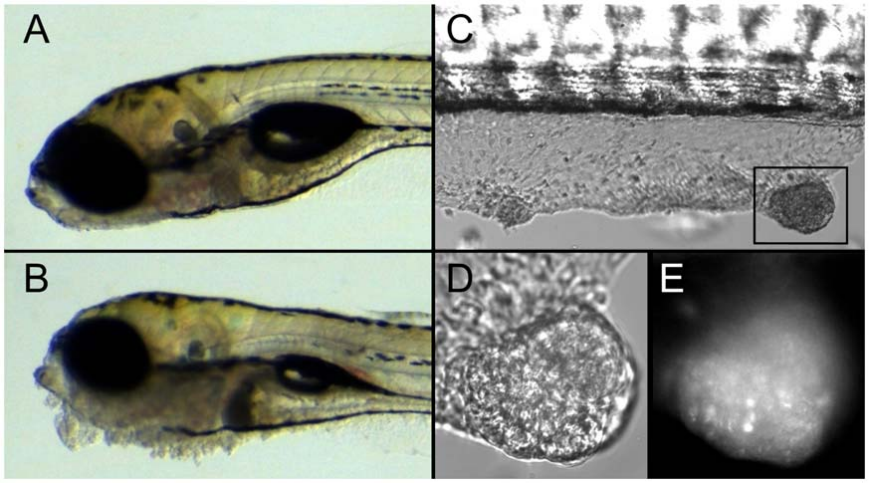
*pen/lgl2* deficient epidermal cells form tumors in a cell autonomous fashion. DIC images of 5-day-old wild-type (A) and *pen/lgl2* mutant larvae (B). DIC image of wt host with *pen/lgl2* mutant skin clones (C). Close-up of a tumor in DIC (D) and GFP channel (E). In comparison to 5 day wild-type larvae (A) the *pen/lgl2* mutant larvae exhibit neoplasias, most prominently in the ventral jaw region (B). Seven days after the transplantation of *pen/lgl2* mutant cells at blastula stage, recipients develop tumor like structures in the skin (C). These tumor like structures (D) contain GFP labelled cells (E) indicating that they are derived from mutant clones.

We have previously shown that zebrafish *pen/lgl2* larvae show overgrowth of epidermal cells (Figure 1A and 1B; and [20]). As *pen/lgl2* mutant larvae die at 4–5 days post fertilization (dpf), it was not clear whether these hyper-proliferating cells would be able to form tumor like structures. To test this, we transplanted cells from *pen*/*lgl2* homozygous mutant donor individuals, into wild type hosts during blastula stage. In order to trace the donor cells in the wild type recipients, donor embryos were transgenic for ubiquitously expressed GFP. The recipient larvae with epidermal cells from mutants or wild type siblings were monitored for potential tumor development. On the 7th day after the transplantation, 17 of the 24 (71%) larvae that had received *pen*/*lgl2* deficient cells (GFP marked) in the epidermis developed epidermal tumors (Figure 1C and 1D). In contrast, none of the larvae that had received cells from wild type siblings developed epidermal tumors (n = 77). Fluorescence microscopic analysis of these tumors revealed that they contain GFP positive cells, indicating that the *lgl2^−/−^* cells are inducing the formation of epidermal tumors (Figure 1E). Interestingly, *lgl2* ^−/−^ cells in other tissues, including brain, did not show any visible hyperplasia at this stage. We conclude that *pen/lgl2* mutant cells are capable of inducing epidermal tumor formation, even if surrounded by wild type tissue. The levels of GFP expression varied in the tumor cells (Figure 1E). This may reflect a variegated expression of GFP. However, we cannot rule out the possibility that some wild type cells also contribute to the neoplastic tissue. Our results support the notion that *lgl2* acts as a tumor suppressor gene specifically in the epidermis.

### Loss of *pen/lgl2* function results in EMT of basal epidermal cells

**Figure 2:**
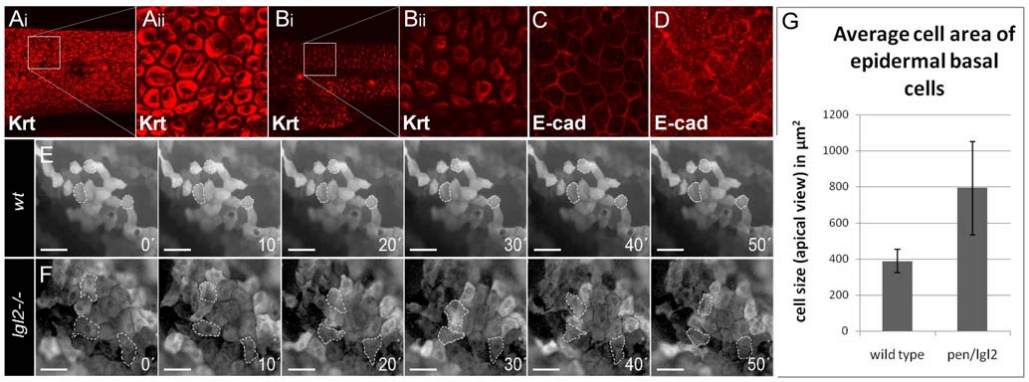
Epidermal cells undergo EMT in *pen/lgl2* mutant larvae. Basal epidermal cells of wild type and *pen/lgl2* mutant 5dpf stained using pan 1–8 Cytokeratin antibody (Ai - Bii) and E-cad antibody (C, D). Time lapse analysis of tg(ΔNp63::Gal4,UAS::GFP) labelled cells in wild-type (E) and *pen/lgl2* mutant larvae (F) at 5dpf. Analysis of cell area in wild-type and *pen/lgl2* mutant larvae (G). In contrast to wild-type larvae (Ai, Aii), in *pen/lgl2* mutant larvae the cells appear spindle shaped with keratin accumulation around the nucleus (Bi, Bii). Furthermore, in wild-type basal epidermal cells (C), E-cad localizes to the cell membrane; cells exhibit perfect polygonal shapes. In *pen/lgl2* mutant larvae (D) membrane localization of E-cad is strongly reduced with concomitant increase in the cytoplasmic fraction. Time-lapse analysis reveals (E, F) that there is no change in the shapes of wild-type epidermal cells (E). However, in *pen/lgl2* mutants (F) the shape of epidermal cells dramatically changes over time, indicating their metastable cell fate. Cells develop lamellipodia like structures, a classic trait exhibited by mesenchymal cell types (see Videos S1, S2, S3, S4 in addition). As epidermal basal cells flatten and develop lamellipodia like cell protrusions in *pen/lgl2*, the cell area in apical view is increased in these larvae (G). However, average area of epidermal basal cells in these mutants is highly variable compared to wild types.

Along with uncontrolled cell proliferation, migratory behavior mediated by EMT is another hallmark of cancer cells [25]. In *pen/lgl2* mutant larvae, epidermal cells not only hyper proliferate but also exhibit different morphological shape as evident from the changes in the keratin organization from basal polygonal in wild type to peri-nuclear and spindle shaped in *pen/lgl2* mutants (Figure 2A and 2B, [20]). Such alterations in cell morphology are indicative of EMT. Since E-cad membrane levels are inversely correlated with acquisition of EMT [26]–[28], we investigated the localization of E-cad in *pen/lgl2* mutants. We found that the membrane localization of E-cad was severely reduced in the mutant epidermis, while the cytoplasmic fraction appeared to be increased compared to the wild type siblings (Figure 2C and 2D).

To observe the behavior of basal epidermal cells of *pen/lgl2* mutants, we generated a transgenic line driving the expression of GFP in basal epidermal cells under the control of Δ*N-p63* promoter [29]. Accordingly, tg(*ΔN-p63::Gal4,UAS::GFP*) zebrafish larvae show GFP expression exclusively in this cell type (Figure S1). To test whether *pen/lgl2* mutant cells acquire migratory properties, we performed time lapse studies of tg(ΔN-p63::Gal4,UAS::GFP)*;lgl2^−/−^* larvae. These revealed that, in contrast to their wild type siblings, basal epidermal cells in *pen/lgl2* mutant larvae dramatically alter their shape, form numerous lamellipodia like structures and exhibit net displacement over time (Figure 2 and Videos S1, S2, S3, S4). From an apical perspective, the observed changes in cell morphology also lead to an increase in average cell area (Figure 2G). The mutant epidermal cells appear larger when compared to wild type cells as they exhibit a highly flattened morphology and develop lamellipodia like cell protrusions.

We conclude that in absence of *pen/lgl2* function the basal epidermal cells lose their epithelial morphology and acquire the morphology of migratory (mesenchymal) cells indicating that these cells undergo the morphological changes associated with EMT.

### Expression profile of *pen/lgl2* mutant larvae reveals differential transcriptional regulation of known molecular regulators of EMT

**Figure 3:**
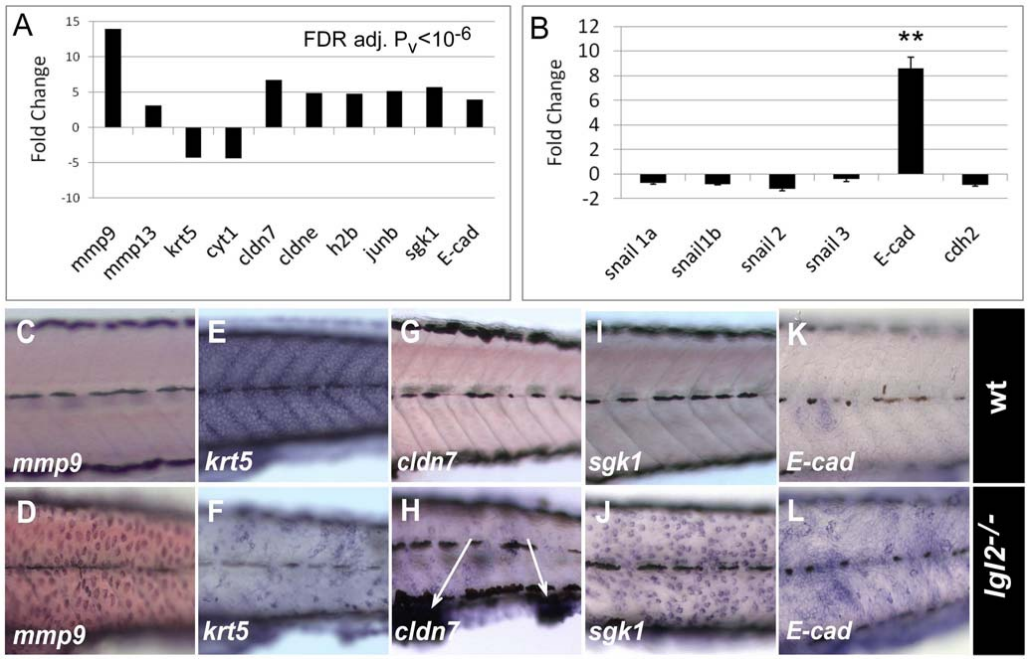
*pen/lgl2* mutant epidermal cells differentially express EMT regulators. Graphical representation of expression data of some of the relevant genes obtained by microarray (A) and quantitative RT-PCR (B). RNA In situ hybridization analysis in wild-type (C, E, G, I, K) and *pen/lgl2* mutant larvae (D, F, H, J, L) at 5dpf. Gene expression data obtained by microarray analysis reveals strong transcriptional activation of EMT associated matrix metalloproteinases like *mmp9* and *mmp13*, cell-cycle related genes like proto-oncogene *jun-b* and histone 2b, whereas expression of cytokeratins like *krt5* or *cyt1* is decreased. Interestingly, tight junction proteins *cldn7*, *cldne* as well as adherens junction component E-*cad* and *serum- and glucocorticoid-induced kinase-1* (*sgk1*) were also up-regulated in *pen/lgl2* mutant. RT-PCR analysis (B) revealed that *E-cad* expression is indeed significantly up-regulated in mutant larvae with marginal decrease in snail expression levels. In situ hybridization analysis (C–L) reveals that the relevant genes are differentially expressed in the epidermis. Note that *cldn7* is highly up-regulated in the cellular clumps in the finfold (arrows in H). ** = P<0.005

To understand if *pen*/*lgl2* mutants also show molecular signatures of EMT and if so, which of the known EMT related genes are active in *pen*/*lgl2* mutant basal epidermal cells, we performed a genome wide expression profiling of *pen/lgl2* mutant larvae showing neoplastic overgrowth at 108hpf using the Agilent microarray platform. Subsequent statistical analysis revealed 117 genes to be significantly differentially regulated in *pen/lgl2* mutant larvae (FDR; P≤10^−6^) (Figure 3A and Table S1). Amongst these differentially regulated genes, we found a very strong transcriptional induction of *mmp*s such as *mmp9* (11.1 fold) and *mmp13* (3.1 fold), which are known regulators of EMT, mainly in the context of malignancy [30]–[32]. Further, we found a set of *cytokeratins*, *krt5*, *ckrt1*, *ckrt2*, and collagens to be down-regulated within a range of 3.5 to 4.4 fold, which is consistent with the previous analysis of the role of cytokeratins in EMT [28]. Moreover, genes involved in cell cycle regulation (e.g. histone-b, jun-b, N-ras), cell survival (e.g. *sgk1*) and tight-junction formation (e.g. *cldn-7*, *cldn-e*, *cldn-c*, *cldn-i*) were also up-regulated from 3.6 to 5.6 fold (Figure 3A).

The canonical way to achieve EMT is to down-regulate *E-cad* at the transcriptional level. The transcriptional repressors, mainly those of the snail family, play an important role in this process [27], [33]–[35]. Intriguingly, we observed robust up-regulation of *E-cad* expression at the mRNA level (Figure 3A). By performing quantitative RT-PCR in *pen/lgl2* mutant larvae, we detected 8 times higher *E-cad* RNA levels in mutants compared to the wild type sibling controls (Figure 3B). Further, examination of E-cad by western blot analysis revealed increase in protein levels in *pen/lgl2* mutant larvae compared to their wild type siblings (data not shown). Thus, although the membrane localization is drastically perturbed (Figure 2C and 2D), E-cad protein levels are higher in *pen/lgl2* mutants. We further estimated the expression levels of *snail* family members. Consistent with up-regulation of E-cad levels, none of the *snail* family members shows increased expression in *pen*/*lgl2* mutants. These data indicate that in *pen/lgl2* mutant larvae EMT of basal epidermal cells is facilitated by mis-localizing E-cad rather than its snail mediated repression.

We confirmed the tissue specificity of differential expression of the genes *mmp9*, *sgk1*, *cldn7*, *krt5* and *E-cad* by in-situ hybridization (ISH) (Figure 3C–3L). We verified the up-regulation of *mmp9* as well as *sgk1* and down-regulation of *krt5* specifically in the basal epidermal cells. Interestingly, the up-regulation of the tight-junction gene *cldn7* was observed mainly in the cells that form epidermal cell aggregates in the ventral jaw region and in the fin-fold (Figure 3H).

Our analyses indicate that basal epidermal cells in *pen/lgl2* larvae exhibit both the morphological and transcriptional characteristics of cells undergoing EMT. We conclude that *pen/lgl2* function is essential to suppress epidermal neoplasia and EMT. Thus, *pen/lgl2* acts as a recessive tumor suppressor gene in vertebrates. Furthermore, canonical snail mediated repression of E-cad is not involved in EMT in *pen/lgl2* mutant. Instead, EMT is facilitated by removal of E-cad from the plasma membrane.

### Inhibitors of ErbB signaling suppress cell proliferation and EMT in basal epidermal cells of *pen/lgl2* mutant larvae

**Figure 4:**
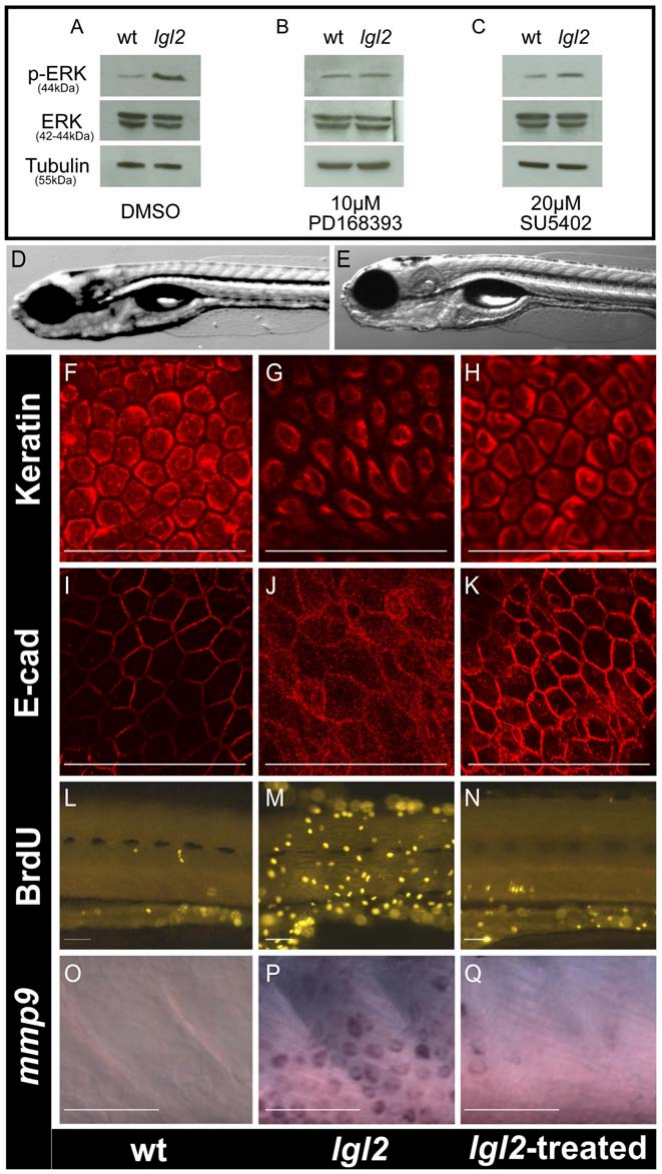
Inhibition of ErbB signaling restores the epidermal morphology and cell cycle in *pen/lgl2* mutant larvae. Western blot analysis of Erk1/2 phosphorylation in untreated wild-type and *pen/lgl2* larvae (A), larvae treated with PD168393 (B) and larvae treated with SU5402 (C). DIC images of wild-type (D) and *pen*/*lgl2* mutant (E) treated with the ErbB inhibitor PD168393 and genotyped. The pan 1–8 cytokeratin antibody staining (F–H), anti E-cad staining (I–K), anti BrdU antibody staining (L–N) and in situ hybridization staining for *mmp9* (O–Q) of the epidermis of wild-type (F, I, L, O) *pen/lgl2* mutant (G, J, M, P) and mutant larvae treated with PD168393 (H, K, N, Q). In comparison to wild-type larvae (A, left lane, 44kD) Erk shows higher level of phosphorylation in *pen/lgl2* mutant larvae (A, right lane 44kD). The levels of Erk phosphorylation are equal in the mutants treated with PD168393 (B) but not with SU5402 (C). The α-Tubulin levels (55kD) are indicative of equal protein loading. The epidermal cell morphology, E-cad localization, cell proliferation and *mmp9* expression in *pen/lgl2* larvae, treated with PD168393 (H, K, N, Q) appears similar to wild-type larvae (F, I, L, O) than the untreated mutant larvae (G, J, M, P). Note that PD168393 treated (rescued) larvae were genotyped to confirm their genotypes.

As human carcinomas are often a consequence of massive over activation of growth factor signaling [36], we assayed *pen/lgl2* mutant larvae for the phosphorylation level of the mitogen activated protein kinase Erk, a common member of growth factor signaling cascades, by western blot [37]. We found elevated levels of phosphorylated Erk in the mutants compared to their wild type siblings (Figure 4A). To identify which growth factor signaling is activated, we treated larvae from heterozygous *pen/lgl2* carriers with inhibitors for three different receptor tyrosine kinases (RTKs), FGFR, IGFR and EGFR, starting at 96 hpf just prior to the appearance of the *pen/lgl2* mutant phenotype. These treatments revealed that an inhibitor of ErbB (PD168393) reduced the levels of phosphorylated Erk (Figure 4B) and rescued the epidermal neoplasia phenotype as well (Figure 4D and 4E). Similar rescue in the epidermal phenotype was observed with another ErbB inhibitor AG1478 (data not shown). In contrast, Inhibition of FGFR (SU5402) neither affected the levels of phosphorylated Erk (Figure 4C) nor did it rescue the epidermal overgrowth phenotype (data not shown). Similarly, there was no rescue in the epidermal overgrowth phenotype when IGFR signaling was inhibited using AG1024 (data not shown). Genotyping of an entire clutch treated with ErbB inhibitor (PD168393) revealed the expected Mendelian proportion (27 out of 120) of *pen/lgl2* homozygous larvae. We further asked whether the EMT and over-proliferation phenotypes were suppressed after treatment with PD168393. Indeed, cytokeratin and E-cad antibody stainings of inhibitor treated *pen/lgl2* mutant larvae were indistinguishable from wild type siblings (Figure 4H and 4K). Consistently, epidermal cell proliferation and *mmp9* transcript levels were strongly reduced (Figure 4L–4N, Figure 4O–4Q). However, hemidesmosomes did not form in PD168393 treated pen/lgl2 mutant larvae as revealed by electron microscopic analysis (Figure S4).

Our data indicate that loss of functional *pen/lgl2* results in over activation of ErbB signaling which promotes over proliferation of basal epidermal cells as well as cellular EMT by transcriptional modulation of EMT regulators. We further conclude that over-activation of ErbB signaling is not the cause for the absence of hemidesmosomes in the *pen/lgl2* mutant. This observation further indicates that disruption of *pen/lgl2* primarily affects hemidesmosome formation, which is consistent with the previous analysis [20],[21].

### *erbB2* promotes EMT but not the cell proliferation in the basal epidermal cells of *pen/lgl2* mutant larvae

**Figure 5:**
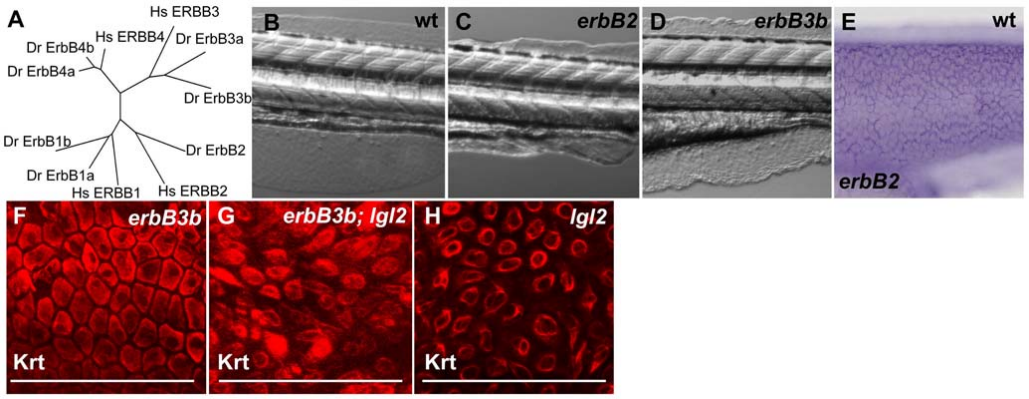
Phylogenetic analysis of *erbB* family members and zebrafish mutants of *erbB* paralogs. (A) Phylogenetic analysis of *erbB* family members in the zebrafish genome (zv7) with the human orthologs (Minimum evolution algorithm, 100 replicates). With the exception of *erbB2*, all other *erbB* family members are duplicated in zebrafish. (B–D) DIC Images of wild-type (B), *erbB2^−/−^* (C) and *erbB3b* ^−/−^ (D) larvae at 132hpf. (E) In-situ hybridization analysis of *erbB2* at 48hpf. Keratin staining in *erbB3b* mutant larva (F) *erbB3b/lgl2* double mutant larva (G) and *pen/lgl2* mutant larva (H) at 132 hpf. Note that in *erbB3B;lgl2* double mutants (G) epidermal cells appear spindle shaped as in *pen/lgl2* mutant larvae (H). *erbB3b* (F) mutations alone do not affect the morphology of basal epidermal cells.

In mammals four ErbB receptors are known, ErbB1 to ErbB4, which get activated upon the binding of ligands such as EGF, HB-EGF, neuregulins, betacellulin [38]. Ligand binding leads to the formation of homo- or heterodimers amongst these receptors, resulting in signal transduction [38]). Our bioinformatic and phylogenetic analysis coupled with the previous analysis of some of the family members [39],[40] revealed that with the exception of *erbB2*, all other members of this family exist in duplicates in the zebrafish (Figure 5A). *erbB2* as well as *pcs/erbB3b* zebrafish mutants exhibit defects in glia development and regeneration [39]–[42]. We found *erbB2* to have an additional epidermal phenotype in the fin-fold (Figure 5C) and it is also expressed in the epidermis (Figure 5E). To determine which of the ErbB receptors is activated in *pen/lgl2*, we performed loss of function studies in a *pen/lgl2* mutant background. We knocked down *erbB1a* with a splice site antisense morpholino. Injections of *erbB1a* morpholino did not interfere with the *pen/lgl2* phenotype but reproduced the cardiovascular phenotype published earlier [43] (data not shown). However, we cannot exclude the involvement of *erbB1a* in promoting the EMT and growth phenotype in *pen/lgl2* mutant larvae as we found the effect of the morpholino to decrease beyond 48hpf, possibly due to dilution effects (Figure S2). Double mutants of *pcs/erbB3b^−/−^,pen/lgl2^−/−^* genotype did not show suppression of the *pen/lgl2* neoplastic phenotype and immuno-histological analysis using the pan 1–8 Cytokeratin antibody did not reveal any reduction in strength and initiation of EMT phenotype (data not shown, Figure 5I). Taken together, these results demonstrate that *erbB3b* function is not essential for neoplasia and EMT phenotype in *pen/lgl2* mutants.

**Figure 6:**
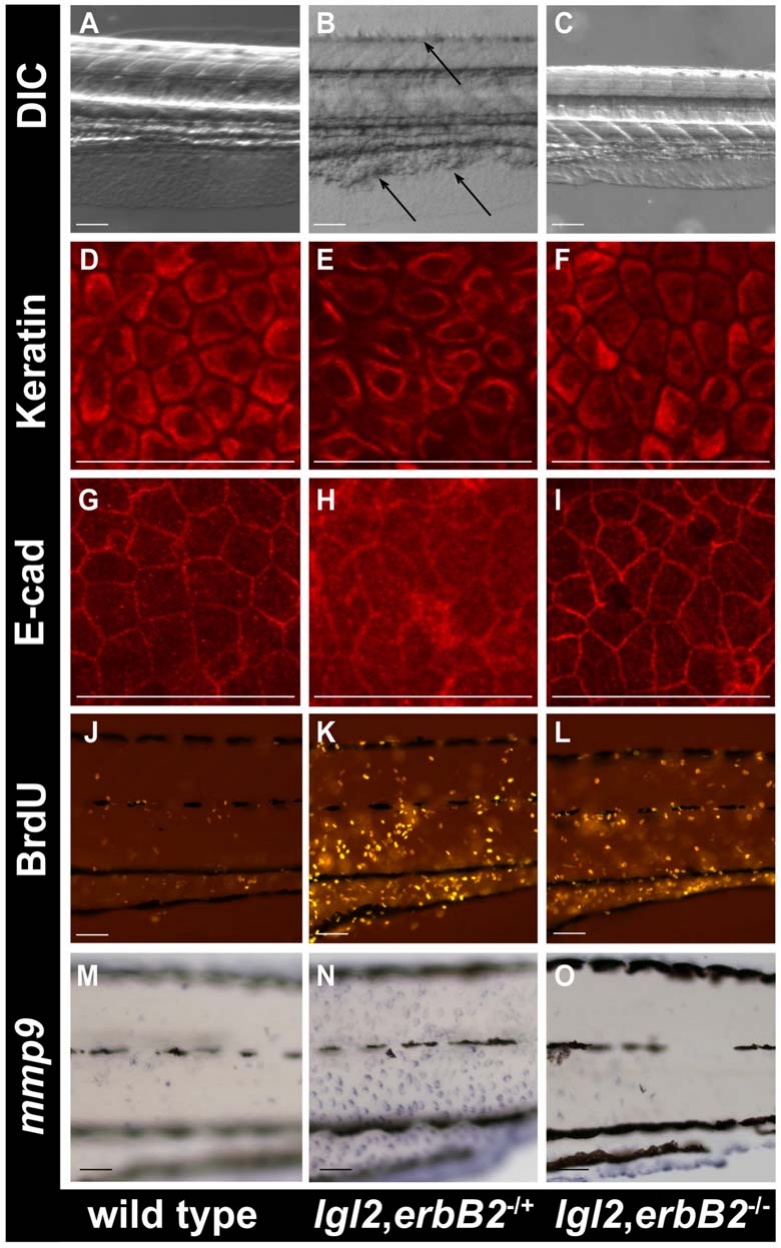
*erbB2* promotes EMT in the epidermis of *pen/lgl2* mutant larvae. DIC images of the morphology of wild-type (A), *lgl2*,*erbB2^+/−^* (B) and lgl2,erbB2^−/−^ (C) larvae at 108hpf. Keratin (D–F), E-cadherin (G–I), BrdU (J–L) and *mmp9* (M–O) staining in wild-type (D, G, J, M), *lgl2*,*erbB2^+/−^* (E, H, K, N) and *lgl2*,*erbB2^−/−^(*F, I, L, O) larvae at 108hpf. The DIC images of the morphology reveals that cellular clumps, a typical characteristic of *pen/lgl2* mutant larvae are present in *lgl2*,*erbB2^+/−^* (B) but are absent in *lgl2,erbB2^−/−^*(C). The keratin, E-cadherin and *mmp9* staining in *lgl2,erbB2^−/−^* (F, I, O) appear similar to wild-type (D, G, M) indicating *erbb2* promotes the EMT phenotype in *pen/lgl2* mutant larvae. Note that, in *lgl2,erbB2^−/−^* larvae (L) BrdU incorporation does not decrease to wild-type levels (J).

In zebrafish, *lgl2* and *erbB2* are both located on chromosome 12 (23.6mb distance). To study double mutants, chromosomal recombinants were made (see Materials and Methods). We investigated progeny from *lgl2* ^−/+^, *erbB2* ^−/+^ double heterozygous fish (3 crosses; n = 296), which were sorted for the morphological epidermal *lgl2* phenotype at 108hpf as well as 132hpf and subsequently genotyped. We identified 40 larvae that were homozygous for both *lgl* and *erbB2*. In 37 of these, no epidermal phenotype was detected up to 132hpf (Figure 6C). In contrast, all *lgl2* larvae with *erbB2* ^+/−^ or *erbB2* ^+/+^ genotype showed strong phenotypes or lethality at the same stage (Figure 6B, Table S2). In a separate experiment, we analyzed Cytokeratin and E-cadherin localization, *mmp9* expression and BrdU incorporation in *lgl2^−/−^*,*erbB2^−/−^* double mutants at 108hpf when the *lgl2* phenotype becomes apparent. The morphology of the epidermal cells in the double mutant larvae, as revealed by Keratin staining, appeared completely normal at 108hpf Figure 6D–6F). Occasionally, epidermal cells in *pen*/*lgl2; erbB2* double mutant larvae exhibited milder changes in Keratin organization, indicative of altered cellular morphology at 132 hpf (data not shown). E-cadherin localization appeared normal in the epidermis of the double mutants (Figure 6G–6I) and *mmp9* expression levels in the epidermis of double mutant larvae were comparable to those in wild type (Figure 6M–6O). Interestingly, however, the BrdU incorporation analysis revealed that (Figure 6J–6L) there is no significant decrease (t-test, p>0.05) in the epidermal cell proliferation in a predefined area in *lgl2^−/−^*,*erbB2^−/−^* double mutants (29±1.0, n = 3) as compared to *lgl2^−/−^*,*erbB2^−/+^* (32±9, n = 3) larvae.

Our data suggest that *erbB2* but not *erbB3b* mediated signaling is responsible mainly for the EMT phenotype in *pen/lgl2* mutant basal epidermis. We did not observe rescue in the epidermal cell-proliferation phenotype in *pen*/*lgl2; erbB2* double mutants and epideramal cell morphology was altered as well at later stages indicating that *erbB2* deficiency doesn't completely rescue the phenotype. Since the inhibitors completely rescue both the proliferation as well as EMT phenotypes, we propose that other ErbB receptors might be involved in promoting epidermal cell-proliferation in *pen/lgl2* mutants.

## Discussion

Impaired cell polarity is one of the hallmarks of carcinoma but was considered to be a secondary effect for a long time. Recent findings however suggest it can also be a cause rather than a consequence of tumor progression [18],[19],[44],[45]. In many epithelia, the apico-basal polarity is established by the activity of the apical aPKC-Par3-Par6 pathway and baso-lateral Lgl-Scrib-Dlg pathway. Surprisingly, only loss of function in baso-lateral pathway components leads to epithelial or brain neoplasia phenotypes in *Drosophila* [45]. In vertebrates, two components of this pathway, *lgl* and *dlg*, have multiple orthologues [45]. Loss of *lgl1* function results in brain hyperplasia in the mouse [17]. In zebrafish loss of *lgl2* function leads to over-proliferation of epidermal cells [20]. Here, we demonstrate that transplantation of *pen/lgl2* homozygous mutant cells during blastula stage into wild type embryos results in the formation of tumor like structures in the epidermis after 7 days. This analysis clearly shows that *lgl2* behaves as a tumor suppressor gene in vertebrates.

The basal epidermal cells in *pen/lgl2* mutants do not form hemidesmosomes and display an altered cell morphology instead of the polygonal cell shape found in wild type larvae [20]. E-cad localization at the plasma membrane is reduced in the mutant epidermal cells indicating that epidermal cells undergo EMT. Indeed, GFP labeling of basal epidermal cells revealed that in *pen/lgl2* mutant larvae these cells show continuous changes in their morphology, project lamellipodia and move as a sheet. This sheet like movement has been described as a metastable cell state in the context of EMT [28]. Consistent with the cellular analysis, the expression profiling of *lgl2* mutant basal epidermal cells revealed differential expression of the known EMT markers and regulators such as *matrix-metalloproteinases* and *keratins*. Intriguingly, the levels of *E-cad* RNA are high in the *pen/lgl2* mutant. This is in contrast to known developmental scenarios of EMT such as the delamination of neural crest cells or gastrulation where E-cad expression is downregulated at the transcriptional level by a transcriptional repressor, snail [34],[35]. In addition, structural components of tight junctions, such as several *claudins*, are also up-regulated in *pen/lgl2* mutants. Thus, the expression profiles of cell adhesion molecules in *pen/lgl2* larvae resemble several human breast carcinomas including an inflammatory breast cancer (IBC), a highly aggressive subtype of human breast cancer, which has been characterized by E-cad and *erbB2* over-expression [46]–[51]. The up-regulation of *claudin genes* is intriguing. However, it is not clear whether Claudins localize properly in the epidermal cells. Even if the Claudins do localize properly, the in-situ expression analysis suggests that *claudins* are up-regulated mostly in the cellular clumps that are formed in the median finfold or in the ventral jaw region. It is thus clear that claudins are not expressed during the process or EMT but rather when the mesenchymal cells re-acquire partial epithelial phenotypes while forming tumor like aggregation. However, further analysis is required to test this notion.

How does deficiency in components of the baso-lateral pathway lead to epithelial cell proliferation and EMT? It has been suggested that the protein Scrib stabilizes the coupling between E-cad and the Catenins and thus behaves as a regulator of epithelial cell adhesion and migration [52]. Our data show that for Lgl2 the mechanism to suppress EMT and tumor formation is fundamentally different. Lgl2 may not manifest its function by stabilizing the coupling between E-cad and Catenins. Previously, we have shown that neither the loss of maternal nor zygotic *lgl2* function primarily affect E-cad localization [20],[21]. Consistent with mammalian data, knockout of *E-cad* in the mouse or zebrafish epidermis does not result in EMT [21], [53]–[55]. This indicates that although the loss of E-cad facilitates EMT [56], it may not be sufficient to induce it in animal models.

We show that inhibition of *erbB2* signaling, either genetically or by small chemical inhibitors, leads to suppression of EMT and a neoplastic phenotype in *pen/lgl2* mutant larvae. Intriguingly, E-cad membrane localization is restored in these larvae, indicating that the loss of E-cad is a consequence of activation of ErbB signaling rather than a cause of it. Thus, our analyses presented here suggest that Lgl2 acts as a tumor suppressor by regulating the amplitude of ErbB signaling in the epidermis. Since we did not observe *snail*-mediated down-regulation of *E-cad*, we propose that an *erbB2* dependent pathway in *pen/lgl2* mutants leads to the destabilization of E-cad at adherens junctions. Indeed in *pen/lgl2* mutant epidermal cells, known modifiers of E-cad function, such as *mmp9* and *sgk*1 are up-regulated (Figure 3). While Mmps are known to be involved in ecto-domain shedding of E-cad [57],[58], Sgk1 functions in the phosphorylation of Ndrg1, a protein involved in vesicular recycling of E-cad [59],[60]. Additionally, recently published data from cell culture suggests an involvement of RTK signaling in the destabilization of adherens junctions via Numb [61]. Here, Numb functions as an adapter protein coupling E-cadherin to the Par polarity-complex. This interaction was shown to be sensitive to elevated levels of RTK signaling, leading to mislocalization of E-cadherin [61]. Further analysis involving loss of function of *mmps* and *sgk1* as well as studies of Numb localization in the *pen/lgl2* mutant larvae would be necessary to clarify their contributions to the EMT phenotype.

The primary function of Lgl2 in hemidesmosome formation [20],[21] is not dependent on ErbB signaling as hemidesmosomes do not form in *pen/lgl2* mutants even after the inhibition of ErbB signaling (Figure S4). This suggests that the loss of hemidesmosomes might be the cause but not a consequence of the activation of ErbB signaling. This hypothesis is supported by the fact that components of hemidesmosomes (e.g. Itga6/Itgb4), physically interact with the ErbB2 receptor tyrosine kinase [62]–[64]. Thus, it is possible that mislocalization of hemidesmosomal components in *pen/lgl2* mutants lead to an activation of ErbB2.

ErbB signaling plays an important role in the development of human carcinomas, as it is able to induce proliferation and EMT and further is able to suppress apoptosis [36],[65]. This is in particular true for ErbB2 which is over-expressed in more than 25% of all breast carcinoma [66]. A causal link between ErbB activation and loss of cell polarity, which is one of the hallmarks of carcinomas, has recently been established. It has been shown that activated ErbB2 associates with Par6-aPKC leading to disruption of the apico-basal polarity [67]. While ErbB2 regulates apico-basal cell polarity by interacting with apical polarity components like Par6-aPKC, our analyses presented here suggest that one of the basolateral pathway components, Lgl2, regulates the activation of ErbB2. Thus, there seems to be a reciprocal interaction between cell polarity regulators and ErbB2 signaling. Interestingly, although ErbB2 activation induces cell proliferation, the association of ErbB2 with Par6-aPKC was not essential for regulating cell proliferation indicating that ErbB2 affect the cell proliferation independent of disruption of apico-basal cell polarity [67]. Our data suggest that at the organismal level loss of ErbB2 does not affect the epidermal cell proliferation phenotype in *lgl2* mutants but only prevents EMT. Since inhibitors, which do not discriminate between various ErbB family members rescue both, proliferation as well as EMT phenotypes, it appears that activation of more than one ErbB family member might be involved in promoting epidermal neoplasia in *pen/lgl2* mutants. Thus, cell proliferation and EMT phenotypes are not coupled in *pen/lgl2* mutant larvae.

The ErbB signaling pathway includes multiple ligands and receptors in vertebrates. ErbB1 and ErbB4 represent discrete RTKs, as they contain ligand binding sites as well as a kinase activity, essential for auto-phosphorylation and signal transduction. In contrast, ErbB2 and ErbB3 do not independently transduce extracellular signals as homodimers since ErbB2 has no ligand-binding domain and ErbB3 has no kinase domain. Thus, ErbB2 and ErbB3 must interact with each other or with other ErbB receptors to transduce signals [38]. Analysis of the *Danio rerio* genome revealed that genome duplication events have led to the duplication of *erbB1*, *erbB3* and *erbB4* making the situation even more complex. We found that homozygous mutations in *erbB2* but not *erbB3b* dramatically rescued the EMT phenotype, establishing ErbB2 signaling to be responsible for the onset of EMT. However, the direct activator of ErbB2, in promoting the EMT phenotype in *lgl2* mutants remains unidentified at this point.

To summarize, *lgl2* function is essential for tumor suppression and EMT in the vertebrate epidermis. *pen/lgl2* mutant cells are able to induce the formation of epidermal tumors when surrounded by wild type cells. In the absence of Lgl2, hemidesmosomes do not form and ErbB signaling is activated, which results in induction of pathways involved in promoting EMT via mislocalization of E-cad as well as proliferation. Amongst several *erbB* paralogues, *erbB2* is more directly involved in transducing the signal. Thus, the *pen/lgl2* mutant would serve as a very good model to perform chemical screens for compounds that are able to suppress *erbB* signaling or important downstream effectors and thus have high potential for cancer therapeutic research.

## Materials and Methods

### Fish strains

The morphological and immuno-histological analysis of *pen/lgl2* mutant was carried out in Tuebingen (TUE) and WIK background. For transplantations, embryos from *pen/lgl2* mutants in the background of an β-actin::GFP transgenic line were used as donors and from *albino* fish as recipients. Live imaging was performed in transgenic ΔNp63::Gal4, UAS::GFP line. The *erbB2* mutant allele used in this work is st61 [39]. The *erbB3b* mutant allele is HJ036, which contains a C to A point mutation at position 156 leading to a premature stop after 50 amino acids [41]. As *lgl2* and *erbB2* are located on the same linkage group, to generate double mutants, we crossed *pen/lgl2^+/−^* fish with *erbB2^+/−^* fish and out-crossed the obtained trans-heterozygote F1 fish to albino fish. The F2 generation were then screened by genotyping for both, the *pen/lgl2* and *erbB2* mutations.

### Phylogenetic analysis

Protein sequences for ErbB zebrafish paralogues and human orthologues were obtained from Genebank (http://www.ncbi.nlm.nih.gov) and through Ensembl (http://www.ensembl.org/Danio_rerio) databases. Alignments were performed using ClustalW. Phylogenetic analysis was run using neighbor joining, maximum parsimony and minimum evolution algorithms, (MEGA4, http://www.megasoftware.net/). All analysis methods showed similar tree morphology with comparable bootstrap values (1000 replicates). Human orthologues (Genebank ID): ERBB1: 1956, ERBB2: 2064, ERBB3: 2065, ERBB4: 2066. Zebrafish paralogues (Ensembl transcript ID): *erbB1a*: ENSDART00000027219, *erbB1b:* ENSDART00000031151, *erbB2:* ENSDART00000003932, *erbB3a:* ENSDART00000014892, *erbB3b:* ENSDART00000049893, *erbB4a:* ENSDART00000092114, *erbB4b:* ENSDART00000100398

### Generating ΔNp63::Gal4, UAS::GFP transgenic fish

The ΔNp63 promoter was cloned by enzymatic restriction of BAC dkey-13d19 with HhaI (NEB) and subsequent blunt-end cloning of a resulting 4.96 kb fragment into a plasmid containing a Gal4,UAS::GFP expression cassette and mini-TOL2 sites (based on [68],[69]). Transgenesis was achieved by simultaneous injection of plasmid DNA and transposase RNA at 1-cell stage using WPI PV830 pneumatic injection system followed by F1 screen for GFP positive larvae.

### BrdU labelling

5-day-old wild type and *pen/lgl2* mutant larvae were incubated with 10 mM BrdU solution in 2% DMSO in embryonic medium (E3) for 2 hours. After treatment, larvae were washed several times in E3 fixed overnight in 4% PFA in PBS at 4°C. Staining was performed as described below.

### Immunohistochemistry

For IHC procedures the following antibodies were used: Anti-BrdU antibody ab6326 (abcam); anti Cytokeratin antibody Ks pan 1–8 (Progen Biotechnik) and anti E-cad antibody (BD Transduction Laboratory). Embryos were either fixed in 4% PFA (E-cad, BrdU) or in Dent's fixative (cytokeratin). After downgrading the larvae to 0.1 M phosphate buffer (PB), they were washed with PBT (PB+0.8% Triton X-100) five times and blocked in 10% normal goat serum. For BrdU staining, larvae were treated with 4 N HCl for 20 min., washed in PB and blocked in 1% BSA for 1–3 hours. Antibodies were diluted as: Ks pan 1–8 (1∶10), anti-E-cad (1∶250), anti-BrdU (1∶50) and samples were incubated at room temperature for 4 hours or overnight at 6–8°C. Afterwards, larvae were washed five times in PBT, incubated with Cy3 or Alexa 488 anti-mouse or anti-rat antibodies, post fixed in 4% PFA and upgraded in 70% glycerol for fluorescence/light microscopy.

### In situ hybridization

DIG-labeled RNA probes for *cldn7*, *krt5*, *mmp9* and *sgk1* were prepared from larval 5dpf total cDNA obtained from *pen/lgl2^−/−^* mutants. In situ hybridization was performed using Intavis in-situ robot (model: insituPro VSi).

Probe templates have been amplified with the following primer combinations by PCR reaction and subsequently cloned into pGEM-T Easy Vector (Promega). DIG-labeled probes were synthesized using T7 and Sp6 RNA polymerases (Roche).

*E-cad*: 5′-TTACTTCTGCTATTGCTTGCT-3′; 5′-TCATAGTCTTGGTCGTTTCCT-3′

*cldn7*: 5′-TGGCACATAAAGGACTGCAA-3′; 5′-CGATGAAAATAGCTGCACCA-3′

*erbB2*: 5′-GTCATCCAGAACGAAGATCAG-3′; 5′-CATCAGTCTCCAGATCTCCA-3′

*krt5*: 5′-CAGGAGCTCAGTGTCCTTCC-3′; 5′-CGGTTGTTGAGGGTCTTGAT-3′

*mmp9*: 5′-GCTGCTCATGAGTTTGGAC-3′; 5′-CCGAGCTTCTCGATTTTACG-3′

*sgk1*: 5′-ATGGAACGACGTCAACCTTC-3′; 5′-GCGTAAGCTTCTTGGCATTC-3′

### Transplantations

Transplantations were carried out at blastula stage. After transplantations, donor and corresponding recipients embryos were cultured together (3–5 in number) in a 24 well plate. The *pen/lgl2* mutant donors were identified at 4.5dpf by phenotype or by molecular genotyping at blastula stage (RFLP). The host larvae that received mutant or wild type clones (GFP-positive) in the skin were sorted and further raised up to 7–8 dpf and analyzed by microscopy for tumor phenotypes.

### Inhibitor treatment

For screening, all inhibitors (SU5402; AG1024; PD168393; AG1478 all Calbiochem) were used at 10–50 µM concentration in 1% DMSO in E3 Medium starting at 96hpf. For BrdU, IHC and ISH experiments, PD168393 and AG1478 were used at a concentration of 10 µM starting at 96hpf.

### Western blot and detection

For western blots, mutants were identified by the development of the characteristic *lgl2* mutant phenotype at 108hpf. Three times 40 mutants and equivalent number of wild type larvae were collected at 108hpf and than treated with either DMSO as a control, or inhibitors for ErbB signaling (10 µM) or FGFR signaling (20 µM) for 12 h. Subsequently, larval tails were collected by cutting posterior to the swimming bladder on ice. Proteins were extracted in DXB (25 mM Hepes, pH 6.8, 50 mM KCl, 1 mM MgCl2, 1 mM DTT, 250 mM sucrose) containing Roche Complete protease inhibitors (Cat No. 11836153001) and Pierce Halt phosphatease inhibitors (Cat No. 78420). Protein concentration was determined using OD at 280 nm (NanoDrop). For PAGE, NuPage 4–12% gradient gels (Invitrogen) were used. After transfer, equal loading was re-checked by poinseau red staining. Antibodies used were: p-ERK (M9692; 1∶600), Erk (M5670; 1∶500), α-Tubulin (T9026; 1∶10000) (all from Sigma). Secondary antibodies used were HRP conjugated anti-mouse (p-Erk, Tubulin) and anti-rabbit (Erk). Detection was performed using chemiluminiscence (ECL^+^; GE Healthcare).

### Microarray analysis

The microarray experiment was conducted using tissues (tail) posterior of the anal opening of wild type (Tuebingen) and *pen* larvae at 5 dpf. RNA for microarray analysis was extracted from four biological replicates using TRIZol Reagent (Gibco BRL, Eggenstein, Germany). Complementary RNA was prepared from 1 µg total RNA from each replicate as described in the Agilent Low RNA Input Linear Amplification kit manual (Agilent Technologies, Palo Alto, CA, USA). Double-stranded cDNA was synthesized using the reagents from this kit, and Cy3- or Cy5-labeled cRNA was prepared by cDNA in vitro transcription in the presence of cyanine 5-CTP or cyanine 3-CTP dyes. Fluorescently labeled RNA was then purified with the Qiagen RNEasy spin columns, according to the manufacturer's protocol (Qiagen, Hilden, Germany). After purification, cRNA was fragmented and used to hybridize to the zebrafish G2519F 4X44 microarray platform containing 4 duplicated arrays of the 22,000 probe-set design (Agilent Technologies, Palo Alto, CA, USA). Four biological replicates were conducted, including two dye swap experiments to minimize the effect of any potential dye bias. Hybridization, washing, and scanning were performed according to the manufacturer's protocol. The microarrays were scanned on a Genepix Axon 4000B scanner (Molecular Devices, Union City, CA, USA) at five micron resolution with five-line averaging. Raw expression values from each probeset were extracted using the Genepix Pro 6.0 feature extraction software, and features were flagged manually for poor quality. The data were then analyzed in the R statistical programming environment using the Bioconductor module Limma [70],[71]. Duplicate probesets on each array were considered as technical replicates for the analysis in addition to the 4 biological replicates on separate arrays. A standard linear model for differential expression, with the Limma module in Bioconductor, was used to identify genes up and down regulated in the mutant versus wild type experiment. The resulting p-values from the hypothesis tests were adjusted for multiple testing with the false discovery approach (FDR) to control for false positives [72]. In addition, the empirical Bayes approach automatically adjusts raw p-values for multiple testing and generates a B-statistic that may also be used for ranking differentially expressed genes [70]. All microarray data may be accessed through the ArrayExpress repository on the European Bioinformatics Institute database website (http://www.ebi.ac.uk/microarray-as/ae/).

### Quantitative real-time PCR

As for the microarray experiment, larval tails posterior to the anal opening were used as tissue sample (3 technical replicates of each biological replicate; 3 biological replicates). Total RNA was isolated using TRIZOL reagent (Invitrogen) and cDNA was synthesized using AMV cDNA Synthesis Kit (Invitrogen) with oligo-dT15 primers (Promega). SYBR Green (BioRad) was used for quantitative real-time PCR. Time emission readings were recorded with DNA Engine Opticon 2 (MJ Research), and analyzed as described [73]. *gapdh* was used for normalization (ΔΔC(t) method) as described [74]. Statistical significance was determined using student's t-test.

### Genotyping of mutant larvae

Genotyping to indentify mutants was done by a PCR based restriction fragment length polymorphism (RFLP) method for *lgl2-* and *erbB2-* or by DNA sequencing for *erbB3b* mutants. The RFLP analysis was done as follows- the mutations in *erbB2* cause a loss whereas in *lgl2* a gain of a restriction site. PCR product from genomic DNA samples obtained by the primer pairs: 5′- ATGCATACCTTCCTGGAGTAG-3′; 5′-TGTGGTTCTAGTGGAGGAGGA-3′ (for *lgl2*) or 5′-TGAAGAATGCTGGTAGCTGG-3′ and 5′-GGACTCAGCAAAGG ACTTAC-3′ (for *erbB2*) was digested with either SfcI (for *lgl2* mutation) or BsrGI (for *erbB2* mutation) resulting in a genotype specific DNA band pattern (Figure S3). The *erbB3b* genotyping was done by scoring for the premature stop at position 156bp by DNA sequencing of a PCR product obtained from the genomic DNA using the primer pair 5′-CGCTCTCCTGTTCCTCTGTG-3′; 5′-ACCCTCTTCCTCCATTGTCC-3′ (Figure S3). In case of PFA fixation, individual genomic DNA samples were sampled before fixation (head tissue). The genotype of larvae used in ISH experiments was scored by the development of the characteristic *lgl2* phenotype.

### Movie processing and image analysis

Time-lapse movies were acquired using AxioVision 4.6 (Zeiss) and compressed in the Codec H264. For encoding in H264, the OSS Virtual Dub was used. Cell area (Figure 2G) was determined using the “outline” tool included in Zeiss AxioVision 4.6 (Zeiss) on three biological replicates each from wild type and *pen/lgl2*. Scale bars indicate equal sizes within one figure.

## Supporting Information

Figure S1 Expression of GFP under the ΔNp63 promoter. Antibody staining of 5.5dpf tg(ΔNp63::Gal4,UAS::GFP) zebrafish larvae using anti Cytokeratin (red) and anti GFP antibody (green). The co-labeling of both antibodies reveals activity of the 4.96 kb upstream promoter element of ΔNp63 exclusively in basal epidermal cells in the skin.
(0.70 MB TIF)

Figure S2 Knockdown of *erbB1a* using morpholinos. For knock-down of *erbB1a*, we used a morpholino targeted to a splice site. To test the activity of this morpholino over time, primers spanning the targeted intron-exon boundary were designed. (A) PCR performed on cDNA from morphant zebrafish larvae at different time points reveals morpholino efficiency. (B) A working morpholino causes splice events to fail at the targeted splice site (red) resulting in larger PCR product. Genomic DNA contamination of the cDNA causes a third, larger, product as the amplicon spans two introns on the genomic template. This analysis reveals that the used morpholino only prevents efficient splicing of *erbB1a* RNA before 48hpf, causing equal amounts of spliced vs. morphant RNA at later stages. Primers used for PCR: 5′-CCACCAACA TCGACTCCTTT-3′; 5′-AAACCTTGAGGTCATCCGAG-3′. Morpholino sequence: 5′-AAATGCTCTTCCTCACCCTCTGAAT-3′.
(0.54 MB TIF)

Figure S3 Genotyping of *lgl2*, *erbB2*, and *erbB3b*. The genotype of larvae presented in this work was scored using PCR-based restriction fragment length polymorphisms (RFLP) for *erbB2* and *lgl2* or by sequencing for *erbB3b*. The mutation in *lgl2* causes an artificial restriction site for SfcI. The mutation in *erbB2* causes a loss of a BsrGI restriction site. Treatment of PCR products from individual genomic samples with either SfcI or BsrGI leads to a genotype specific DNA band pattern in agarose gel electrophoresis. The *erbB3b* mutation was scored by sequencing of a PCR product spanning the site of lesion. The nature of the mutation is a cytosine to adenine transversion leading to a premature stop codon after 156bp.
(0.66 MB TIF)

Figure S4 Inhibition of *erbB* signaling does not restore hemidesmosome formation in *pen/lgl2* mutants. *pen/lgl2* mutant basal cells are unable to form hemidesmosomes, even after inhibition of ErbB signaling. EM cross section through larval skin 5dpf reveals hemidesmosome formation at the basal membrane in wild types (A, arrows) whereas *pen/lgl2* mutants (B) and *pen/lgl2* mutants treated with ErbB inhibitor PD168393 (C) lack these structures.
(1.58 MB TIF)

Table S1 Expression profile of *pen/lgl2* compared to wild type. Using microarray technique the expression profile of wild-type versus mutant zebrafish larval tails, posterior to the anal opening was analysed. A significance threshold of adj. p-value of 10^−6^ (FDR) resulted in 117 genes to be significantly differentially expressed. Within those, most prominent genes, involved in EMT and cell cycle, as well as cytoskeleton rearrangements, can be found. Additionally, genes involved in the formation of tight and adherens junctions are present. The down-regulated genes are indicated in red in this table whereas up-regulated genes are indicated in black.
(0.03 MB XLS)

Table S2 Comparison of phenotypes and genotypes in *erbB2,lgl2* double mutant incrosses. Percental distribution of epidermal neoplasia in *pen/lgl2* single- and *lgl2,erbB2* double mutants. Note that the loss of *erbB2* strongly reduces the formation of the characteristic overgrowth phenotype in the *pen/lgl2* mutant background, even at late time points.
(0.03 MB DOC)

Video S1 Phenotype of basal epidermal cells in the wild-type larvae. A 90-minute timelapse movie of tg(*Np63::Gal4,UAS::GFP*) wild-type zebrafish larva at 5dpf. Settings: 20×1-min interval, 10 frames per second. The GFP labelled basal epidermal cells in the wild-type larvae remain static.
(1.81 MB MOV)

Video S2 Phenotype of basal epidermal cells in the *pen/lgl2* mutants larvae. A 90-minute timelapse movie of tg(*Np63::Gal4,UAS::GFP*) *lgl2* ^−/−^ zebrafish larva at 5dpf. Settings: 20×1-min interval, 10 frames per second. The GFP labelled basal epidermal cells exhibit migratory behavior in the mutant larvae. Cells show cell shape changes and development of lamellipodia like cell protrusions, indicating their mesenchymal character.
(6.63 MB MOV)

Video S3 Phenotype of basal epidermal cells in wild-type larvae. A 240-minute timelapse movie of tg(Δ*Np63::Gal4,UAS::GFP*) wild-type zebrafish larva at 5 dpf. Settings: 25×1-min interval, 10 frames per second. The GFP labelled basal epidermal cells remain static in the wild-type larvae.
(9.33 MB MOV)

Video S4 Phenotype of basal epidermal cells in *pen/lgl2* mutant larvae. A 240-minutes timelapse movie of tg(*Np63::Gal4,UAS::GFP*) *lgl2* ^−/−^ zebrafish larva at 5dpf. Settings: 25×1-min interval, 10 frames per second. The GFP labeled basal epidermal cells exhibit migratory properties in the mutant larva. Cells show cell shape changes and formation of lamellipodia like cell protrusions indicating their mesenchymal character.
(13.28 MB MOV)
