# Supplementary figures and images for: Lgl2 Executes Its Function as a Tumor Suppressor by Regulating ErbB Signaling in the Zebrafish Epidermis
Source: PLoS Genet. 2009 Nov 13;5(11):e1000720. doi: 10.1371/journal.pgen.1000720 (PMC2771016; doi:10.1371/journal.pgen.1000720)

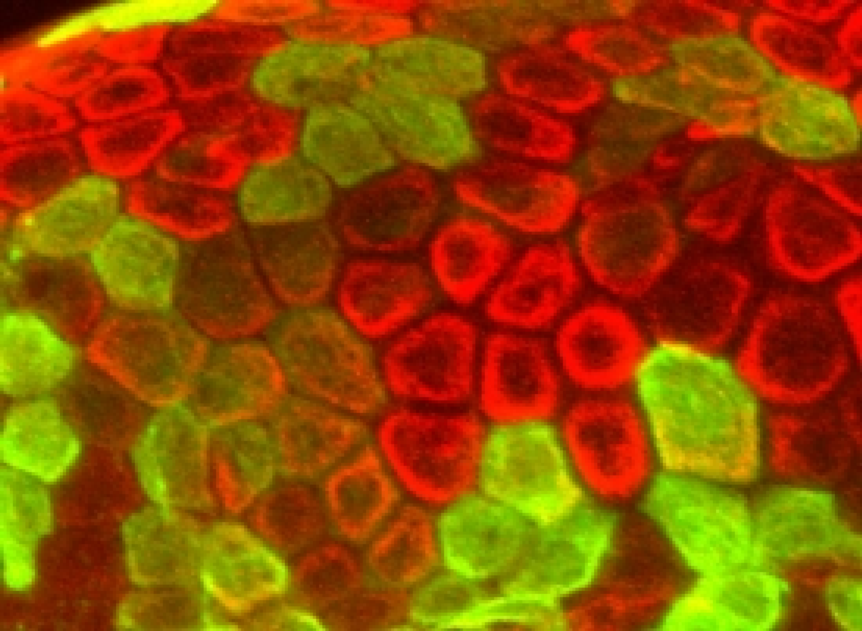

Supplement: Figure S1 — Expression of GFP under the ΔNp63 promoter. Antibody staining of 5.5dpf tg(ΔNp63::Gal4,UAS::GFP) zebrafish larvae using anti Cytokeratin (red) and anti GFP antibody (green). The co-labeling of both antibodies reveals activity of the 4.96 kb upstream promoter element of ΔNp63 exclusively in basal epidermal cells in the skin. (0.70 MB TIF) [file pgen.1000720.s001.tif]

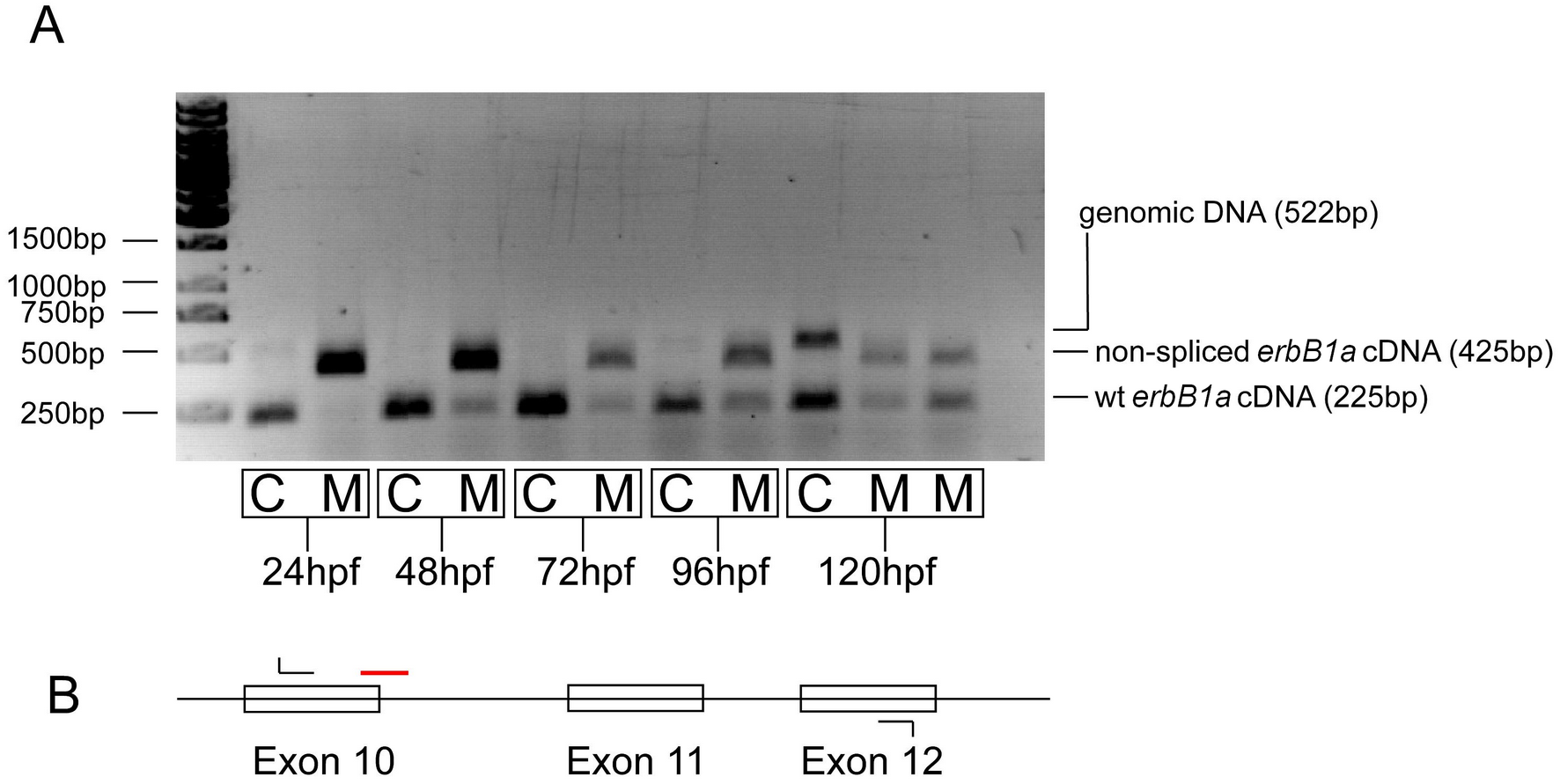

Supplement: Figure S2 — Knockdown of erbB1a using morpholinos. For knock-down of erbB1a, we used a morpholino targeted to a splice site. To test the activity of this morpholino over time, primers spanning the targeted intron-exon boundary were designed. (A) PCR performed on cDNA from morphant zebrafish larvae at different time points reveals morpholino efficiency. (B) A working morpholino causes splice events to fail at the targeted splice site (red) resulting in larger PCR product. Genomic DNA contamination of the cDNA causes a third, larger, product as the amplicon spans two introns on the genomic template. This analysis reveals that the used morpholino only prevents efficient splicing of erbB1a RNA before 48hpf, causing equal amounts of spliced vs. morphant RNA at later stages. Primers used for PCR: 5′-CCACCAACA TCGACTCCTTT-3′; 5′-AAACCTTGAGGTCATCCGAG-3′. Morpholino sequence: 5′-AAATGCTCTTCCTCACCCTCTGAAT-3′. (0.54 MB TIF) [file pgen.1000720.s002.tif]

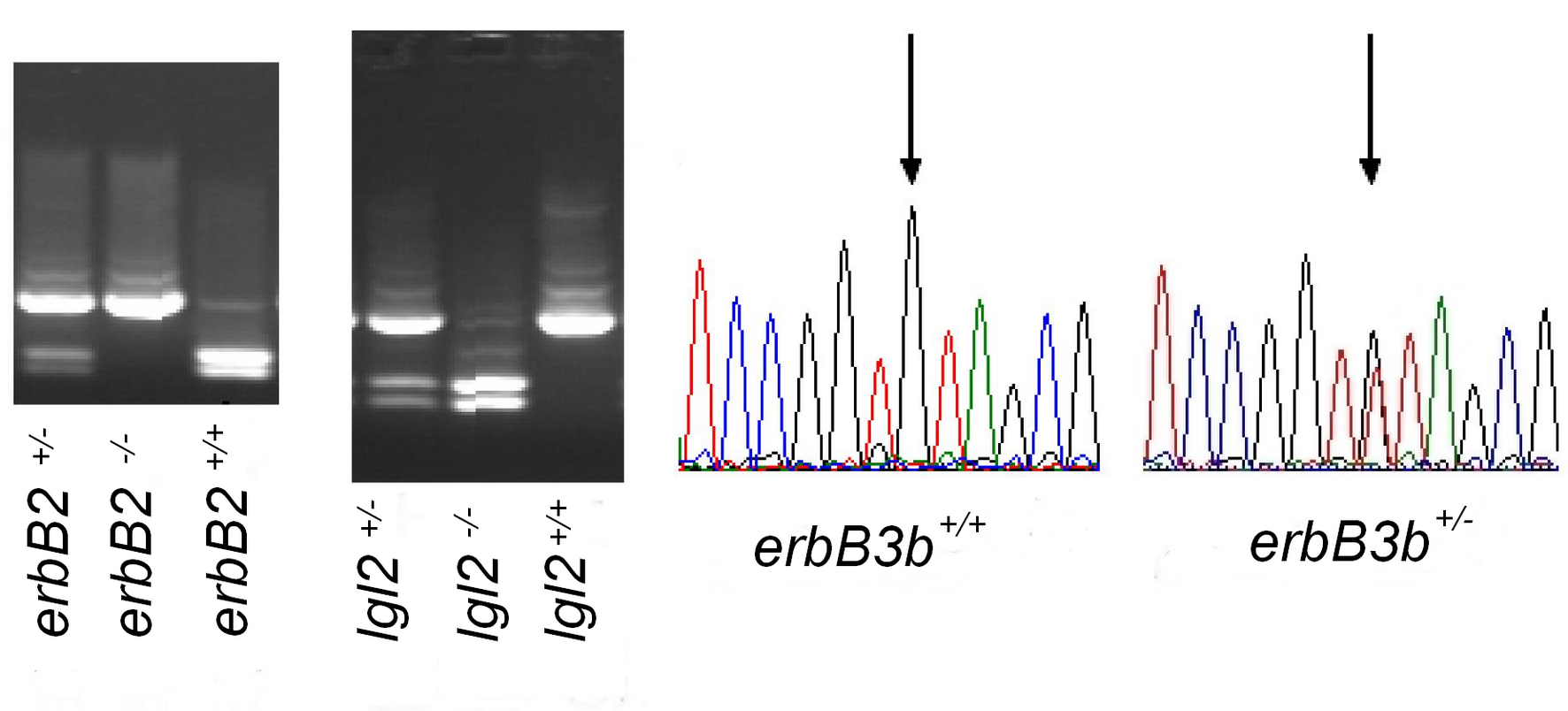

Supplement: Figure S3 — Genotyping of lgl2, erbB2, and erbB3b. The genotype of larvae presented in this work was scored using PCR-based restriction fragment length polymorphisms (RFLP) for erbB2 and lgl2 or by sequencing for erbB3b. The mutation in lgl2 causes an artificial restriction site for SfcI. The mutation in erbB2 causes a loss of a BsrGI restriction site. Treatment of PCR products from individual genomic samples with either SfcI or BsrGI leads to a genotype specific DNA band pattern in agarose gel electrophoresis. The erbB3b mutation was scored by sequencing of a PCR product spanning the site of lesion. The nature of the mutation is a cytosine to adenine transversion leading to a premature stop codon after 156bp. (0.66 MB TIF) [file pgen.1000720.s003.tif]

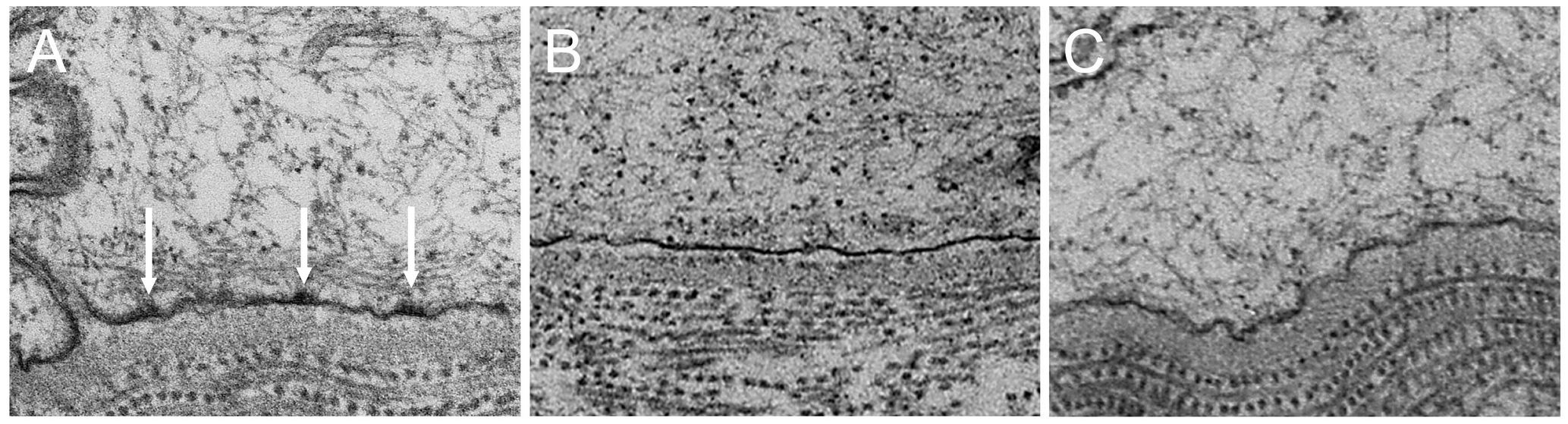

Supplement: Figure S4 — Inhibition of erbB signaling does not restore hemidesmosome formation in pen/lgl2 mutants. pen/lgl2 mutant basal cells are unable to form hemidesmosomes, even after inhibition of ErbB signaling. EM cross section through larval skin 5dpf reveals hemidesmosome formation at the basal membrane in wild types (A, arrows) whereas pen/lgl2 mutants (B) and pen/lgl2 mutants treated with ErbB inhibitor PD168393 (C) lack these structures. (1.58 MB TIF) [file pgen.1000720.s004.tif]
