# Supplementary material for: Lgl2 Executes Its Function as a Tumor Suppressor by Regulating ErbB Signaling in the Zebrafish Epidermis
Source: PLoS Genet. 2009 Nov 13;5(11):e1000720. doi: 10.1371/journal.pgen.1000720 (PMC2771016; doi:10.1371/journal.pgen.1000720)
Supplement: Table S2 — Comparison of phenotypes and genotypes in erbB2,lgl2 double mutant incrosses. Percental distribution of epidermal neoplasia in pen/lgl2 single- and lgl2,erbB2 double mutants. Note that the loss of erbB2 strongly reduces the formation of the characteristic overgrowth phenotype in the pen/lgl2 mutant background, even at late time points. (0.03 MB DOC) [file pgen.1000720.s006.doc]

Table S2

| Phenotype/Genotype | *lgl2^-/-^* | *erbB2^-/-^* | *lgl2^-/-^;erbB2^-/-^* |
| --- | --- | --- | --- |
| overgrowth 108 hpf | 91.4% | 0% | 7.5% |
| overgrowth 132 hpf | 100% | 0% | 7.5% |
